# Inhibition of glycolysis and stimulation of mitochondrial biogenesis lead to increased ROS levels and cell death in HNF-1ß positive clear cell carcinoma

**DOI:** 10.1038/s41419-025-08243-2

**Published:** 2025-12-01

**Authors:** Naoki Kawahara, Hiroki Kuniyasu, Shiori Mori, Shingo Kishi, Sumire Sugimoto, Tomoka Maehana, Shoichiro Yamanaka, Ryuji Kawaguchi, Fuminori Kimura

**Affiliations:** 1 Department of Obstetrics and Gynecology, Nara Medical University, Kashihara, Japan; 2 Department of Molecular Pathology, Nara Medical University, Kashihara, Japan; 3 Department of Cancer Biology, Institute of Biomedical Science, Kansai Medical University, Hirakata, Japan; 4 Department of Pathology, Kansai Medical University, Hirakata, Osaka Japan

**Keywords:** Ovarian cancer, Targeted therapies

## Abstract

Ovarian clear cell carcinoma is characterized by HNF-1ß overexpression and is known to be resistant to chemotherapy. An inhibitor screening that specifically targets HNF-1ß led us to identify Actinonin as a candidate for cancer treatment. Actinonin, which is known to inhibit aminopeptidase M, has also been recognized for its antibacterial properties. We confirmed that GSK-3ß interference/inhibition, as a component of the HNF-1ß pathway, combined with Actinonin, has a highly potent antitumor effect compared to monotherapy. The same effect was observed in renal clear cell carcinoma lines expressing HNF-1ß. Actinonin promoted mitochondrial production by suppressing aerobic respiration, which decreased AMPK levels and increased ROS production. However, it also elevated GADD45α expression and induced mitophagy. GSK-3ß inhibition suppressed glycolysis and shifted energy production to OXPHOS, leading to increased ROS production. Furthermore, this combination produced excess ROS beyond metabolic capacity, which accumulated in lipid bilayers, leading to a further increase in CHOP gene expression and suppression of mitochondrial turnover. The GSK-3ß inhibitor and Actinonin combination demonstrated a powerful tumor-suppressive effect in vivo without severe side effects. Combining GSK-3ß inhibition with Actinonin can effectively eliminate cancer cells with HNF-1ß overexpression by inhibiting glycolysis and promoting mitochondrial turnover, highlighting new options for cancer therapy.

## Introduction

Ovarian cancer is a type of cancer that affects women and is responsible for causing the sixth-highest number of cancer-related deaths [1]. It is often referred to as the “silent killer” [2] because it can be difficult to detect in its early stages. As a result, most cases of ovarian cancer are diagnosed when the disease has already progressed to later stages [3]. Unfortunately, over 185,000 people die every year around the world due to this disease [4]. Ovarian cancer is classified into different subtypes, which include clear cell carcinoma (CCC), endometrial carcinoma, serous carcinoma, and mucinous carcinoma. In recent years, molecular targeted therapy has made significant progress in treating ovarian cancer. PARP inhibitors have proven to be highly effective against serous carcinomas with BRCA1/BRCA2 mutations. However, CCC is resistant to existing chemotherapy, and there is currently no effective molecular-targeted chemotherapy available to treat it. Therefore, it is necessary to explore new methods to improve the treatment outcomes of CCC.

It is widely known that CCC exhibits overexpression of two proteins: hepatocyte nuclear factor 1beta (HNF-1ß) and mitochondrial superoxide dismutase (SOD2) [5]. For example, HNF-1ß impacts the cell cycle, resulting in cell cycle arrest, and promotes survival in human CCC cell lines. This is achieved by enhancing the phosphorylation of the Chk1 protein in response to genotoxic stress [6]. HNF-1ß overexpression also assists in cell survival by maintaining persistent Chk1 activation, which is facilitated by USP28-mediated Claspin stabilization [7]. The overexpression of HNF-1ß contributes to cancer cell survival by phosphorylating glycogen synthase kinase 3 beta (GSK-3ß) and NFκB [8]. The HNF-1ß-GSK-3ß-p-NFκB axis responds to DNA damage, and the inhibition of GSK-3ß suppresses tumor cell growth. In this context, the effect of HNF-1ß overexpression on gluconeogenesis, glycogen accumulation, and aerobic glycolysis is crucial for promoting cell proliferation and survival by upregulating oxidative stress tolerance. Previous studies have shown that HNF-1ß plays a role in promoting gluconeogenesis, glycogen accumulation, and aerobic glycolysis. This leads to an improved tolerance for oxidative stress [9].

The Warburg effect is a phenomenon where cancer cells convert glucose to lactate through pyruvate, even when there is enough oxygen available [10]. This effect is observed in many cancer cells. Reactive oxygen species (ROS) are mainly produced by mitochondrial respiration in the intracellular environment. Cancer cells suppress mitochondrial respiration to avoid ROS accumulation and survive the stressful environment [11]. Therefore, cancer cells depend on aerobic glycolysis to promote proliferation under this strategy to minimize oxidative stress. Normal cells produce energy efficiently by converting glucose to pyruvate, followed by the TCA cycle and electron transport chain (ETC) in mitochondria. It is hypothesized that GSK-3ß could be a master regulator in metabolic alternation cancer cells, particularly in CCC. In this article, we conducted screenings to identify candidates that are specifically effective in targeting the HNF-1ß-GSK-3ß-p-NFκB axis. We will report a unique recipe for the treatment of HNF-1ß overexpression CCC.

## Material and methods

### Cell lines

The TOV-21G(RRID: CVCL_3613) and ES2(RRID: CVCL_3509) cell lines were obtained from the American Type Culture Collection (ATCC) based in Manassas, VA, USA. The cell line 786-O (RRID: CVCL_1051) was kindly derived from the urology department, and RMG-I (RRID: CVCL_1662) was kindly given by H. Itamochi (Tottori University School of Medicine, Yonago, Japan). The cells were maintained in Dulbecco’s Modified Eagle’s Medium/Ham’s F-12 or RPMI with L-Glutamine and Phenol Red. The medium contained 10% fetal bovine serum and 100 U/mL penicillin and streptomycin. Cells were replaced periodically to avoid contamination and kept separate from other cell lines for passages, and regularly checked for Mycoplasma infection.

### Inhibitor screening

The Screening Committee of Anticancer Drugs (SCADS) inhibitor kit III (ver. 1.5), which contains 367 kinase inhibitors, was provided by The Ministry of Education, Culture, Sports, Science and Technology, Japan. We carried out inhibitor library screening. The TOV-21G and ES2 cell lines were grown in 96 plates at a concentration of 5000 cells per well for 24 h, and applied by 10 µM of inhibitors as a final concentration. Dimethyl sulfoxide (DMSO) was applied in the control well. At 48 h after application, we measured cell viability by MTS assay (CellTiter 96® AQueous One Solution Cell Proliferation Assay, Promega, WI, USA) according to the recommended protocol.

### Inhibitors and short interference

We used AR-A014418 (S7435, Selleck chemicals, Houston, TX, USA) and SB216763 (S1075, Selleck chemicals, Houston, TX, USA) as GSK-3ß inhibitors, as well as Actinonin (AG-CN2-0161, Adipogen Life Sciences, San Diego, CA, USA) as a peptide deformylase (PDF) inhibitor. We use si-GSK-3ß (D-003010-09, Horizon Discovery, Cambridge, UK) and si-control (D-001206-14, Horizon Discovery, Cambridge, UK). We used mito-TEMPO (S9733, Selleck chemicals, Houston, TX, USA) as ROS scavenger.

### Double stain of viable or dead cells

We utilized a double staining kit (CS01, Dojindo, Kumamoto, Japan) that contained Calcein-AM and Propidium Iodide (PI). Twenty-four hours after interfering with GSK-3ß (#D-003010-09, Horizon Discovery, CO, USA) (15 nM) or control (#D-001206-14, Horizon Discovery, CO, USA), we administered Actinonin and collected cells, including the supernatant, at 54 h after Actinonin administration. We washed the cells multiple times and mixed them with 2 µM of Calcein-AM and 4 µM of PI, incubating them at 37 °C for 15 min. We then observed both living cells, stained yellowish-green fluorescent, and dead cells, stained red fluorescent, using an excitation filter at 490 ± 10 nm under a fluorescence microscope. We also used a 545 nm emission filter to observe only dead cells.

### Cell cycle and apoptosis analysis

Twenty-four hours after transfection or inhibitor administration, Actinonin was added at 50 µM. Then, the cells were harvested for 48 h and washed in phosphate-buffered saline (PBS) before fixation in cold 70% ethanol, which was added dropwise to the pellet while vertexing. Cells were fixed for 30 min at 4 °C. Fixed cells were washed twice in PBS and centrifuged at 250 × *g* for 5 min. Cells were incubated with 50 µL of a 100 µg/mL stock of RNase and 200 µL propidium iodide (PI) (from 50 µg/mL stock solution). A BD FACSCalibur (RRID:SCR_000401, BD, Franklin Lakes, NJ, USA) flow cytometer was used to analyze the cell population for cell cycle changes.

### Apoptosis assay

The TOV-21G cell were transfected grown in 96 plates at a concentration of 5000 cells per well. After applying Incucyte® Caspase-3/7 Dye (4440, Sartorius AG, Göttingen, Germany; diluted 1:1000) and Incucyte® Annexin V Red Dye (4641, Sartorius AG, Göttingen, Germany; diluted 1:200), cell proliferation and apoptosis were studied using the IncuCyte ZOOM™ Live Cell Imaging system (Essen BioScience) as previously described for kinetic monitoring of proliferation and cytotoxicity of cultured cells [8, 12]. They were transferred to the IncuCyte ZOOM™ Live Cell Imaging system and incubation continued over 100 h. In this incubation time, IncuCyte captured images every three hours. After defining the area of the cells, all images were chronologically analyzed focusing on confluence (%).

### Mitochondrial analyses

The assay group was si-GSK-3ß plus Actinonin, si-control plus Actinonin, and si-control plus DMSO. At 24 h after transfection, Actinonin or DMSO was applied. At 24 or 48 h of incubation time after the inhibitor application, to detect mitochondrial mass, mitophagy, and lysozyme, live cells were incubated with 0.1 µM MitoBright LT Red (MT11, Dojindo, Kumamoto, Japan), 100 nM and 1 µM Mtphagy Dye and Lyso Dye (MT02 and LY02, Dojindo, Kumamoto, Japan), and 1 µM of DAL green (D675, Dojindo, Kumamoto, Japan) to detect autophagy. To assess the hydroxy radical level in mitochondria, we used the Cell MeterTM Mitochondrial Hydroxyl Radical Detection Kit (16055, AAT Bioquest, Pleasanton, CA, USA) as a hydroxy radical detector according to the manufacturer’s protocol, and MitoBright ROS Deep Red (MT16, Dojindo, Kumamoto, Japan) as a mitochondrial superoxide detector, also following the manufacturer’s instructions. Fluorescence images were obtained and quantified by BZ-X710 or BZ-X810 (Keyence, Osaka, Japan). Cytosolic GSH/GSSG quantification was conducted (G257, Dojindo, Kumamoto, Japan) according to the manufacturer’s protocol.

### Real time PCR

RNA extraction was performed at 24 and 48 hafter transfection by a TaqMan Gene Expression Cells-to-CTTM Kit (AM128, Invitrogen, Carlsbad, CA, USA) according to the manufacturer’s protocol. PCR was performed on a CFX Connect Real-Time PCR System (Bio Rad, Hercules, CA, USA) with 4 µL of cDNA, 10 µL of TaqMan Gene Expression Master Mix (4369016, Applied Biosystems, Waltham, MA, USA), 1 µl of c-Myc (Hs00153408_m1, Applied Biosystems, Waltham, MA, USA), PGC-1*α* (Hs00173304_m1, Applied Biosystems, Waltham, MA, USA), AMPK (Hs00272166_m1, Applied Biosystems, Waltham, MA, USA), CHOP (Hs00358796_g1, Applied Biosystems, Waltham, MA, USA), GADD45 alpha (Hs00169255_m1, Applied Biosystems, Waltham, MA, USA) and GAPDH (Hs99999905_m1, Applied Biosystems, Waltham, MA, USA) and 5 µL of nuclease-free water (B-003000-WB-100, DharmaconTM, Cambridge, UK), and analyzed by the relative quantitative method.

### Western Blotting

TOV-21G was grown in a six-well dish (2.0 × 10^5^ cells per well), then we extracted protein at 24, 36, and 48 h after transfection. Samples were resolved by Cell Lysis Buffer (9803S, Cell Signaling Technology, Danvers, MA, USA) and protein was prepared at 500 µg/ml using TaKaRa BCA Protein Assay Kit (T9300A, Takara Bio, Shiga, Japan). Samples were applied to Mini-PROTEAN® TGXTM Gels 4–15% and transferred by Trans-Blot® TurboTM Transfer Pack (BIO-RAD, Hercules, CA, USA) by 5 µg. The following antibodies were used for western blotting: primary antibodies against HNF-1ß (RRID: AB_399805, 612504, BD, Franklin Lakes, NJ, USA; diluted 1:1000), m-TOR (7C10) (RRID:AB_2105622, #2983, Cell Signaling Technology, Danvers, MA, USA; diluted 1:1000), phospho-m-TOR (Ser2448) (RRID:AB_330970, #2971, Cell Signaling Technology, Danvers, MA, USA; diluted 1:5000), AMPK alpha (RRID:AB_330331, #2532, Cell Signaling Technology, Danvers, MA, USA; diluted 1:1000), phospho-AMPK alpha (Thr172) (40H9) (RRID:AB_331250, #2535, Cell Signaling Technology, Danvers, MA, USA; diluted 1:1000), GSK-3ß (D5C5Z) (RRID:AB_2636978, #12456, Cell Signaling Technology, Danvers, MA, USA; diluted 1:1000), PINK1 (D8G3) (RRID: AB_11179069, #6946, Cell Signaling Technology, Danvers, MA, USA; diluted 1:1000), Parkin (D4Z1W) (#32833, Cell Signaling Technology, Danvers, MA, USA; diluted 1:1000), phospho-Parkin (Ser65) (#36866, Cell Signaling Technology, Danvers, MA, USA; diluted 1:1000), TFAM (RRID: AB_10841294, #7495, Cell Signaling Technology, Danvers, MA, USA; diluted 1:1000), TOM20 (D8T4N) (RRID: AB_2687663, #42406, Cell Signaling Technology, Danvers, MA, USA; diluted 1:2000), and ß-actin (13E5) (RRID: AB_2223172, #4970, Cell Signaling Technology, Danvers, MA, USA; diluted 1:10,000). Horseradish peroxidase-conjugated secondary antibodies against rabbit and mouse (RRID: AB_631746, sc-2004 and RRID: AB_2687626, sc-516102, Santa Cruz Biotechnology, Dallas, TX, USA; diluted 1:10,000) were used.

### Seahorse XFe96 metabolic flux analysis

The Seahorse Extracellular Flux (XFe96) analyzer (Seahorse Bioscience, MA, USA) was used to determine the real-time oxygen consumption rates (OCRs) and extracellular acidification rates (ECARs). Using XFe96 cell culture plates, 1.0 × 10^4^ cells of TOV-21G per well were seeded and transfected overnight with a complete medium. After 24 h of incubation, cells were washed in pre-warmed XF assay media containing 2 mM glutamine, pH 7.4 for ECAR measurements, or in XF assay media supplemented with 10 mM glucose, 1 mM Pyruvate, two mM L-glutamine, and adjusted to 7.4 pH for OCR measurements. Cells were then kept in 175 µL/well of XF assay media at 37 °C, in a non-CO2 incubator for 1 h. During the incubation time, 1.0 µM oligomycin, two µM FCCP, 0.5 µM rotenone, and antimycin A were loaded for OCR measurements in XF assay media into the injection ports in the XFe96 sensor cartridge. The measurements were normalized by cell proliferation determined by MTS assay.

### In vivo assay

All animal experiments were conducted according to the Guidelines for Proper Conduct of Animal Experiments (1 June 2006, Science Council of Japan) and this study was approved by the animal ethics committee of Nara Medical University (no. 13436, 13436-1, 13436-2). To generate murine peritoneal tumors, 7.5 × 10^6^ TOV-21G cells in 200 µL of PBS were injected into the abdominal cavity in five-week-old athymic female nude mice (BALB/cSlc-*nu/nu*) (SLC, Japan). The sample size was calculated by the result of our previous study [8]. AR-A014418 was injected as 4 mg/kg with 100 µl of corn oil and Actinonin as 20 mg/kg with PBS and Lipocapsulater FD-S PE (16004641, Hygieia bioscience, Osaka, Japan) under 50% capitalize efficacy. At day three after the tumor cell injection, we separated the mice into two groups: a target (n = 5) and a control (n = 5), randomly. Randomization was performed based on the weight of the mice immediately before drug administration. Reagents were injected every day for 15 days. Two days after the last injection, the mice were sacrificed. The selection criteria for mice were as follows. Mice were selected for the following criteria: tumor formation in the abdominal cavity, weight loss of less than 20% during the course of the study, and no other apparent abnormalities in appearance or behavior. One mouse in the control group was dropped because tumor cells did not grow on it. Laboratory examinations were outsourced (SRL, Tokyo, Japan), and for inspection value limits, the limit values were used as numerical values. During the experiment and/or when evaluating the results, the investigator was not blinded to group assignment.

### Immunohistochemistry

Perform antigen inactivation and blocking treatment. Remove endogenous peroxidase, add 500-fold diluted Ki-67 primary antibody (RRID: AB_443209, ab15580, abcam, Cambridge, England), and leave overnight. After washing, add secondary antibody (RRID: AB_628497, sc-2357, Santa Cruz Biotechnology, Dallas, TX, USA; diluted 1:25) and DAB (415172, Nichiei Bioscience, Tokyo, Japan), and observe under a microscope. A random 10 fields of view were selected at high magnification (×400) and the percentage of stained cells relative to the total was calculated.

### Statistical analysis

Data are presented as mean ± SD for normal distribution and median (range) for non-parametric distribution. Analyses were performed by SPSS v. 25.0 (RRID: SCR_002865, IBM SPSS, IL, USA). A student’s t-test was applied to assess the difference between the target and control groups under a parametric distribution. In the case of multi-variables, the one-way ANOVA test was used, followed by Tukey’s honestly significant difference test was conducted. On the other hand, in the case of non-normal distribution, the Mann–Whitney U test was used for two-group comparisons, and the Kruskal-Wallis test was used for multiple-group comparisons. In figures, those displaying significant differences in normal distribution are marked with square brackets, and those displaying significant differences in non-normal distribution are marked with double arrows. Two-sided p < 0.05 was considered to indicate a statistically significant difference.

## Results

### Screening for candidate inhibitors harboring synthetic lethal effect with HNF-1ß overexpression in CCC

**Fig. 1:**
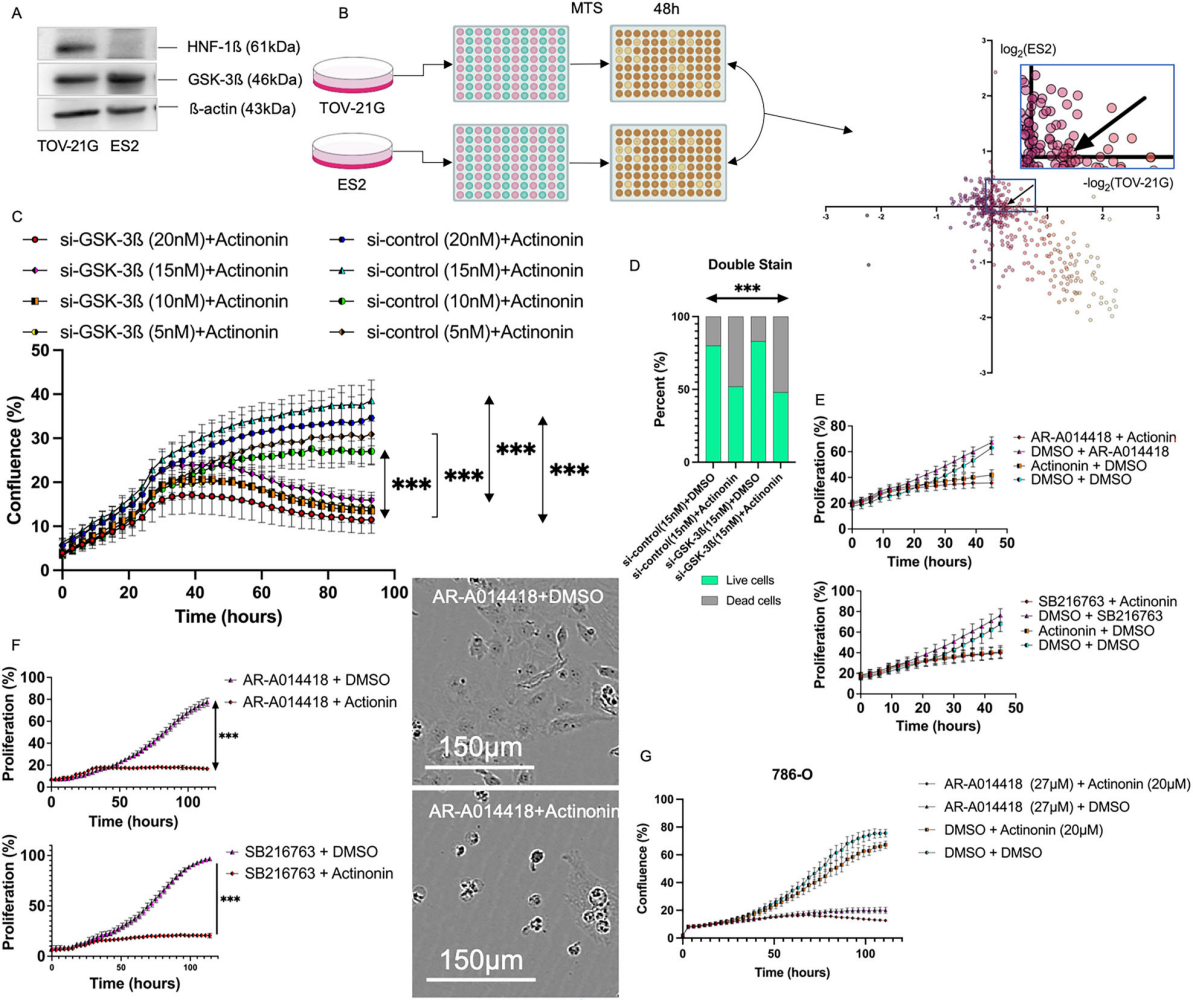
Intense effects on HNF-1ß expressing cell lines by GSK-3ß interference plus Actinonin. **A** The basal protein expression level of HNF-1ß and GSK-3ß for each cell line. **B** There are results from an inhibitor screening comparing TOV-21G (HNF-1ß positive) and ES2 (HNF-1ß negative), where the horizontal axis displays the minus logs of cell proliferation of TOV-21G and the vertical axis displays the logs of ES2. (N = 3) **C** The result of continuous imaging of cell proliferation under Actinonin (50 µM) plus GSK-3ß interference, comparing GSK-3ß interference by each concentration (5–20 nM). (N = 10) **D** Double stains of live and dead cells were analyzed 48 h after Actinonin (50 µM) or DMSO administration plus GSK-3ß or control interference. (N = 3) **E** The results of continuous imaging of cell proliferation under the influence of Actinonin (50 µM) as compared to two GSK-3ß inhibitors, namely AR-A014418 (20 µM) or SB216763 (20 µM). (N = 10) **F** This graph shows the longer continuous imaging result of each GSK-3ß inhibitor with Actinonin (50 µM). The GSK-3ß inhibitor is administered 24 h after seeding, and then seven hours later, Actinonin (50 µM) is added without washing. The upper image is AR-A014418 with DMSO, and the bottom is AR-A014418 plus Actinonin at the last continuous live cell imaging time. (N = 9 or 10) **G** The combination of Actinonin (20 µM) with GSK-3ß inhibitor AR-A014418 (27 µM) was tested on a cell line other than ovarian clear carcinoma, specifically 786-O, which is a HNF-1ß positive renal clear cell carcinoma. (N = 3). The error bars represent ±SD, and those displaying significant differences in normal distribution are marked with square brackets. Those displaying significant differences in non-normal distribution are marked with double arrows. HNF-1ß, hepatocyte nuclear factor-1 beta; GSK-3ß, glycogen synthase kinase 3 beta; DMSO, dimethyl sulfoxide; ***, p < 0.001.The error bars represent ±SD, and p values were calculated using a paired t-test.

Two CCC cell lines were used: TOV-21G, which overexpresses HNF-1ß, and ES2, which is negative for HNF-1ß. Figure 1A shows the protein expression of both. Method outline is in Fig. 1B. Among 46 inhibitors that restricted TOV-21G proliferation, we selected Actinonin as an aminopeptidase M inhibitor. Actinonin (10 µM) inhibited TOV-21G proliferation by 84.5% and 30.0% versus ES2. However, no significant difference was observed between TOV-21G and ES2 in the dose-response curve (Fig. S1A). The cell proliferation assay demonstrated that HNF-1ß interference, combined with Actinonin, resulted in a significant decrease, without dose dependence (p < 0.001) (Fig. S1B). We previously reported that GSK-3ß is downstream of HNF-1ß, then we assessed the tumor-suppressive effect of GSK-3ß interference with Actinonin [8]. The cell proliferation assay showed that GSK-3ß interference combined with Actinonin halted proliferation and caused a decline without dose dependence, with all concentrations resulting in a significant decrease (p < 0.001) (Fig. 1C). Figure 1D shows the cellular impact. Double staining revealed significant differences, indicating that Actinonin induced cell death (p < 0.001). ES2, a negative HNF-1ß cell line, initially inhibited proliferation, but this effect quickly diminished due to GSK-3ß interference with Actinonin (Fig. S1C). AR-A014418 and SB216763, GSK-3ß inhibitors shown to have anti-tumor effects [13], treated cells achieving a growth plateau (AR-A014418 20 µM, SB216763 20 µM, Actinonin 50 µM) (Fig. 1E). Prolonged application of Actinonin completely halted proliferation when combined with AR-A014418 or SB216763 (p < 0.001) (Fig. 1F). To evaluate the effect on another cancer cell line, 786-O renal clear cell carcinoma (HNF-1ß positive) was treated with Actinonin (20 µM) and AR-A014418 (27 µM), leading to a decline and complete destruction of cells (Fig. 1G). The antitumor effect of combining GSK-3ß inhibitor with Actinonin produced similar results at all concentrations (Fig. S1D). We also confirmed that Actinonin mimics GSK-3ß inhibition in RMG-I, a clear cell carcinoma cell line that expresses HNF-1ß. Conversely, we found no effect on cell proliferation in human immortalized epithelial cells (hIEECs) (Fig. S1E). Therefore, while Actinonin or GSK-3ß inhibitors alone allow cell proliferation, their combination can completely stop and decrease it.

**Fig. 2:**
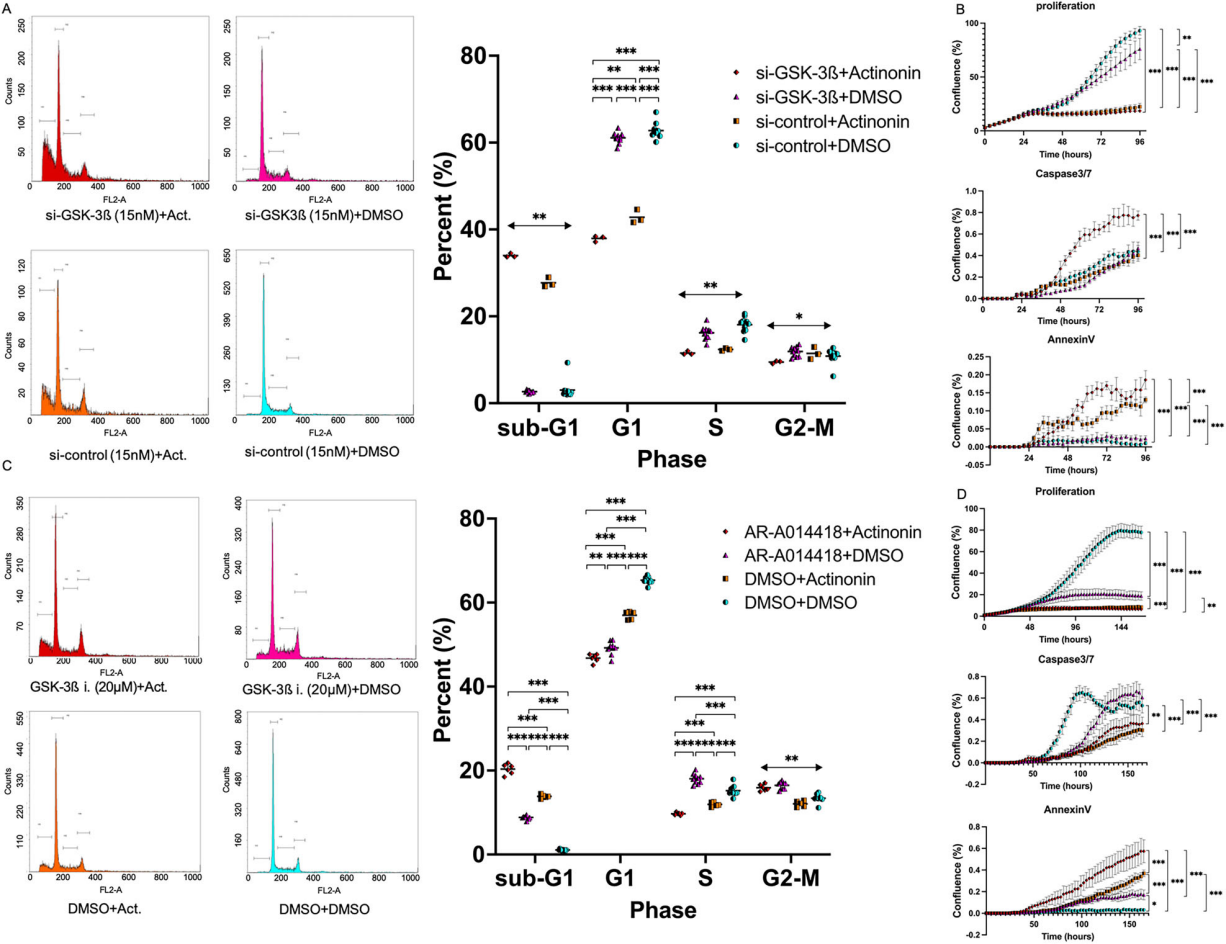
The effects of GSK-3ß interference or inhibitor plus Actinonin on cell cycle and apoptosis. **A** A cell cycle assay was carried out. After 12 h of GSK-3ß interference or control at 15 nM, Actinonin (50 µM) or DMSO was administered after 24 h. Then, 48 h later, the cells were evaluated using a flow cytometer. (N ≥ 3) **B** To determine apoptosis, fluorescence analysis included caspase-3/7 and annexin V were conducted for a long period at 400 nm and 800 nm. Cells were treated with GSK-3ß interference or control at 15 nM, followed by Actinonin (50 µM) or DMSO administration. (N = 10) **C** A cell cycle assay was performed using GSK-3ß inhibitor as AR-A014418 instead of short interference. Actinonin (50 µM) or DMSO was administered at 12 h after seeding. The cell cycle was analyzed 48 hours after the last agent administration. (N ≥ 3) **D** Apoptosis assay was also conducted, as shown in Fig. 3b. After seeding, AR-A014418 (20 µM) or DMSO was administered, and 12 h later, Actinonin (50 µM) or DMSO was added. The cells were then incubated for 48 h. (N = 10). The error bars represent ±SD, and those displaying significant differences in normal distribution are marked with square brackets. Those displaying significant differences in non-normal distribution are marked with double arrows. GSK-3ß, glycogen synthase kinase 3 beta; DMSO, dimethyl sulfoxide; *, p < 0.05; **, p < 0.01; ***, p < 0.001.

### Interference of GSK-3ß Affects Cell Cycle and Apoptosis

Actinonin (50 µM) significantly increased the sub-G1 phase proportion compared to the DMSO group (p < 0.01; both si-GSK-3ß [15 nM] and si-control [15 nM]). Actinonin also significantly decreased the G1 phase proportion versus the DMSO group (p < 0.001; both si-GSK-3ß and si-control), and si-GSK-3ß plus Actinonin decreased it compared to si-control plus Actinonin (37.9 ± 0.6 vs. 42.8 ± 1.5, p = 0.002) (Fig. 2A). These results suggest that si-GSK-3ß plus Actinonin induces strong apoptosis. We conducted an apoptosis assay with caspase 3/7 and annexin V using continuous imaging, where si-GSK-3ß plus Actinonin significantly raised caspase 3/7 levels (p < 0.001). In the Annexin V assay, Actinonin also increased Annexin V levels compared to others (p < 0.001; both si-GSK-3ß and si-control). Similar to the cell cycle assay, si-GSK-3ß exhibited higher annexin V levels than si-control in the presence of Actinonin (p < 0.001) (Fig. 2B). We chose AR-A014418 as the GSK-3ß inhibitor to evaluate its effect with Actinonin on the cell cycle. Results aligned with previous findings, but AR-A014418 (20 µM) alone suppressed the G1 phase (Fig. 2C). In contrast to the short interference study, the proportion of caspase 3/7 positive cells decreased significantly under Actinonin exposure compared to DMSO. In the Annexin V assay, similar to the short interference study, Actinonin increased Annexin V levels compared to the others (Fig. 2D).

### The impact of GSK-3ß interference plus Actinonin on mitochondria dynamics

**Fig. 3:**
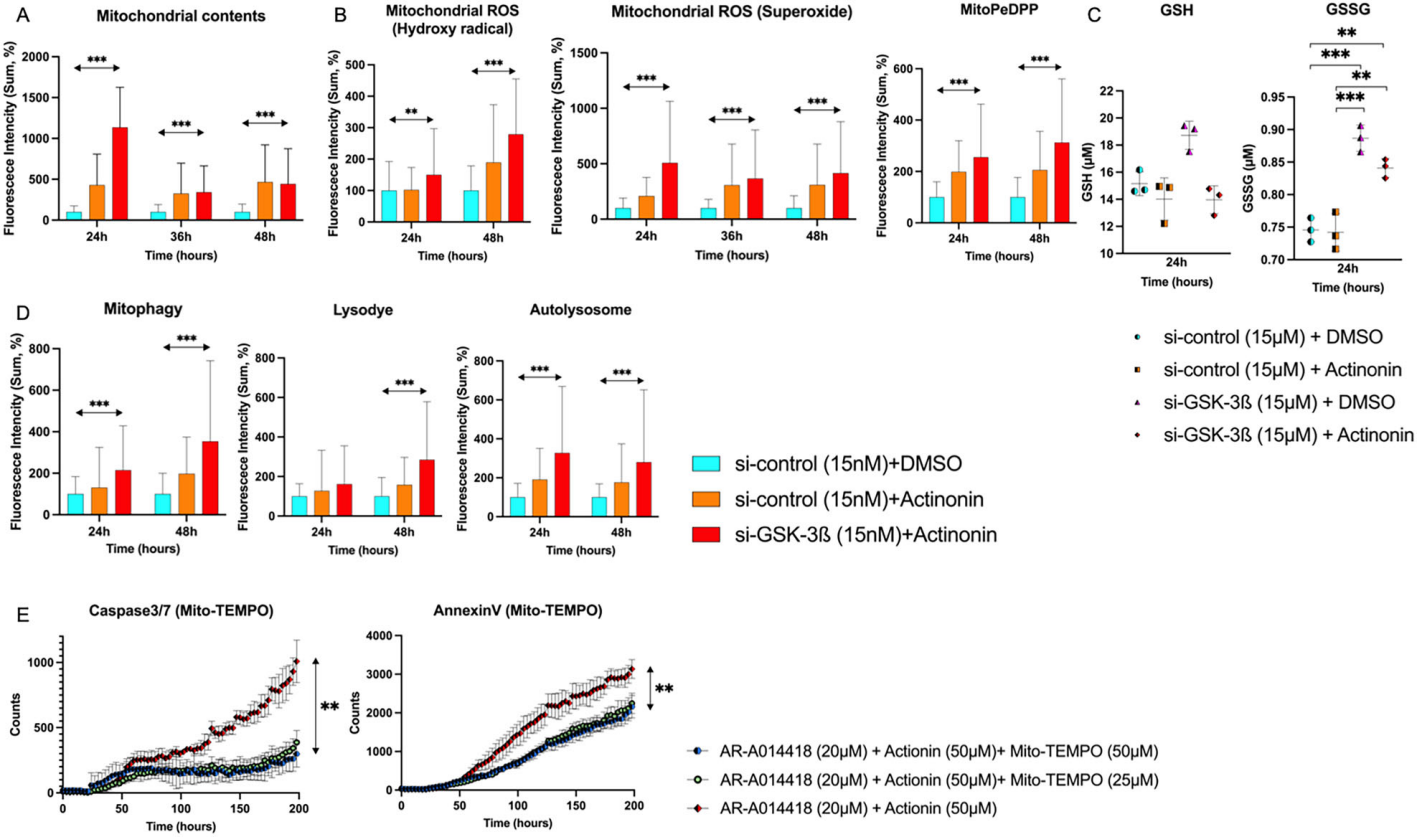
Mitochondrial and ROS dynamics with GSK-3ß interference plus Actinonin. **A** This experiment used a fluorescence assay to measure the contents of mitochondria. The study compared control results to Actinonin (50 µM) alone and Actinonin (50 µM) with GSK-3ß interference (15 nM). (N = 3) **B** The Hydroxyl radical and superoxide status of mitochondria were compared using three different combinations, with and without GSK-3ß interference (15 nM) or Actinonin (50 µM). And to assess the status of mitochondrial fatty acid peroxides, the lipophilic peroxide in living cells under a fluorescence microscope. (N = 3) **C** Antioxidant levels were calculated, specifically GSH (Glutathione-SH) as the reduced form, and GSSG (Glutathione-S-S-Glutathione) as the oxidized form with and without GSK-3ß interference (15 nM) or Actinonin (50 µM). (N = 3) **D** We evaluated mitophagy and autophagy. (N = 3) **E** To assess whether these ROS can be metabolized by ROS scavengers such as MitoTEMPO, and whether they can reverse the caspase-3/7 activation and Annexin V positivity induced by the drug combination. (N = 3). The error bars represent ±SD, and those displaying significant differences in normal distribution are marked with square brackets. Those displaying significant differences in non-normal distribution are marked with double arrows. GSK-3ß, glycogen synthase kinase 3 beta, DMSO dimethyl sulfoxide, PeDPP diphenylpyrenylphosphine-conjugated alkyltriphenylphosphonium iodide; *, p < 0.05; **, p < 0.01; ***, p < 0.001.

After 24 h of Actinonin (50 µM) and GSK-3ß interference (15 nM), mitochondrial intensity additively increased compared to control (335.5 [10.9–1749.2], 491.0 [23.7–1.3 × 10^4^], vs. 81.6 [18.9–445.0], p < 0.001]). At 36 and 48 h, levels were elevated with Actinonin. Still, they did not increase further with GSK-3ß interference (219.0 [54.6–2887.3], 243.8 [32.3–1458.3], vs. 84.4 [13.8–743.4], p < 0.001; 316.0 [37.9–3076.5], 309.4 [27.4–2362.1], vs. 70.8 [17.5–678.5], p < 0.001, respectively) (Fig. 3A). To understand ROS dynamics, we measured hydroxy radical and superoxide. At 24 and 48 hours, hydroxyl radicals increased additively (78.4 [24.1–441.3], 102.7 [13.7–747.1], vs. 73.3 [14.6–792.2], p = 0.003; 141.0 [14.5–1355.1], 241.7 [10.3–945.0], vs. 71.7 [24.7–368.6], p < 0.001, respectively). Similarly, superoxide levels increased in Actinonin monotherapy and combined treatment with Actinonin and GSK-3ß interference compared to the control, but the additive increase was weaker than that of hydroxyl radicals (160.9 [11.3–869.2], 409.8 [2.9–3138.4], vs. 74.4 [5.3–551.6], p < 0.001; 181.4 [23.0–2585.9], 237.7 [27.3–3046.4], vs. 77.2 [10.8–584.4], p < 0.001; 182.9 [30.2–2314.8], 224.9 [37.7–2292.8], vs. 64.8 [18.3–811.8], p < 0.001, respectively). (Fig. 3B). Oxidized MitoPeDPP increased additively in Actinonin monotherapy and in combined treatment with Actinonin and GSK-3ß interference compared to the control (172.4 [14.5–592.4], 176.6 [27.8–879.7], vs. 87.0 [21.6–384.0], p < 0.001; 168.6 [15.7–956.4], 230.4 [24.4–1322.2], vs. 74.8 [11.7–518.1], p < 0.001, respectively) (Fig. 3B). To understand antioxidant dynamics, we measured glutathione concentrations. GSK-3ß interference increased GSH (19.1 [17.5–19.4]), while the combination treatment showed no significant change (14.3 [12.8–14.7]). The oxidized form, GSSG, was higher with GSK-3ß interference plus DMSO or Actinonin compared to the control (0.88 ± 0.02, p < 0.001; 0.84 ± 0.01, p < 0.01) (Fig. 3C). Mitophagy increased additively in Actinonin monotherapy and combined treatment with Actinonin and GSK-3ß interference compared to the control (90.7 [9.7–1478.7], 144.1 [17.8–979.3], vs. 80.3 [9.7–430.8], p < 0.001; 134.2 [17.3–1592.5], 230.7 [20.3–1901.8], vs. 69.9 [15.6–924.7], p < 0.001, respectively). DAL Green, a small fluorescent molecule, detects autophagosomes and autolysosomes. Then the autophagy level increased additively (132.5 [23.3–883.9], 217.7 [46.3–2125.0], vs. 83.1 [24.7–491.2], p < 0.001; 107.7 [24.0–1423.2], 138.1 [22.3–2228.9], vs. 76.8 [25.9–340.7], p < 0.001, respectively) (Fig. 3D). We confirmed that these ROS scavengers, such as MitoTEMPO, can reverse the caspase-3/7 activation and Annexin V positivity induced by the drug combination (Fig. 3E). GSK-3ß interference or Actinonin stimulates ROS metabolism. While Actinonin can independently metabolize ROS, the combination with GSK-3ß interference leads to increased ROS metabolism that surpasses tolerance levels, resulting in ROS accumulation in lipid bilayers and hindering mitochondrial regeneration.

### The energy dynamics by application of actinonin with GSK-3ß interference

**Fig. 4:**
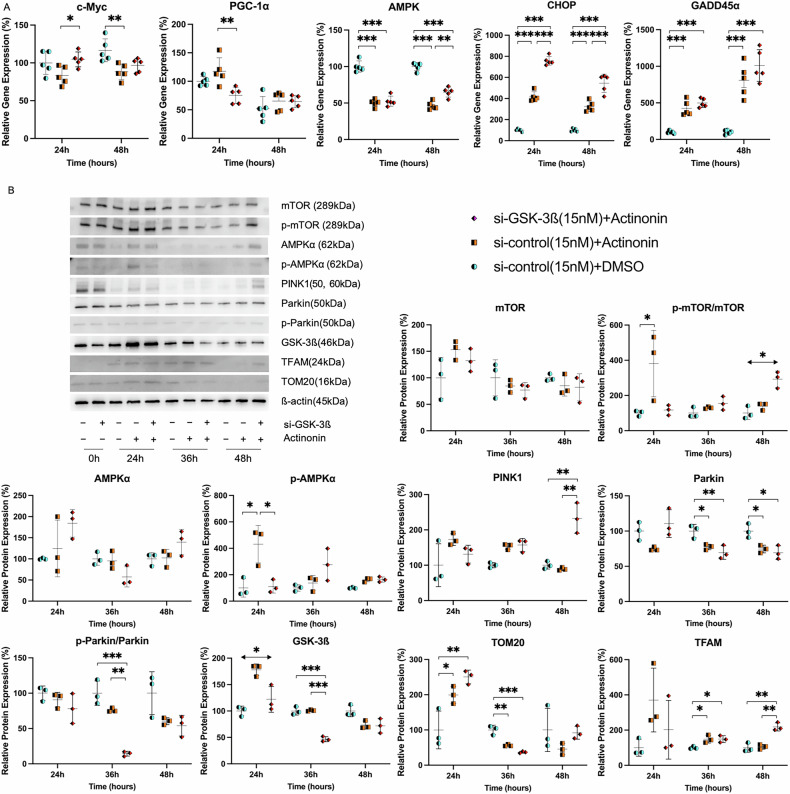
GSK-3ß interference alongside Actinonin affects energy dynamics and mitochondrial stress. **A** The energy dynamics and mitochondrial stress were analyzed by comparing Actinonin (50 µM) alone and Actinonin (50 µM) with GSK-3ß interference (15 nM) to the control. At 24 h after rapid transfection of si-GSK-3ß or control (15 nM), Actinonin or DMSO was added. The cells were then incubated for 24 and 48 h. (N = 5) **B** The impact of GSK-3ß interference combined with Actinonin on mitochondrial membrane proteins. We also assessed the protein transportation ability of mitochondria and mitochondrial DNA. After rapid transfection of si-GSK-3ß or control (15 nM), Actinonin (50 µM) or DMSO was added at 24 h. The cells were then incubated for 24, 36, and 48 h to study the protein expression. (N = 3). The error bars represent ±SD, and those displaying significant differences in normal distribution are marked with square brackets. Those displaying significant differences in non-normal distribution are marked with double arrows. PGC-1α Peroxisome proliferator-activated receptor gamma, coactivator 1 alpha, AMPK AMP-activated protein kinase, CHOP C/EBP homologous protein, GADD growth arrest and DNA damage inducible, m-TOR mechanistic Target of rapamycin, PINK1 PTEN-induced putative kinase 1, GSK-3ß glycogen synthase kinase 3 beta, TFAM mitochondrial transcription factor A, TOM20 Translocase of outer mitochondrial membrane 20, DMSO dimethyl sulfoxide, *, p < 0.05; **, p < 0.01; ***, p < 0.001.

The expression of c-Myc mRNA significantly decreased at 48 h with Actinonin (50 µM) compared to the control (88.2 ± 10.3 vs. 116.6 ± 15.1, p = 0.007). PGC-1*α* expression was notably reduced with GSK-3ß interference and Actinonin compared to Actinonin alone (117.6 ± 23.5 vs. 75.1 ± 14.1, p = 0.004). AMPK levels decreased at 24 h in the Actinonin group (50.0 ± 5.0 and 52.4 ± 6.9 vs. 100.0 ± 7.7, p < 0.001), and at 48 h, levels were lower in the si-control with Actinonin and higher in si-GSK-3ß with Actinonin compared to control with Actinonin (46.5 ± 5.4 and 64.9 ± 6.7 vs. 100.0 ± 5.4, p < 0.001 and p = 0.001). CHOP expression was elevated with Actinonin and GSK-3ß interference at both 24 and 48 h compared to control (p < 0.001). Actinonin also increased GADD45 alpha expression (p < 0.001) (Fig. 4A). No significant difference was observed in m-TOR expression, but phosphorylation showed higher values in the combination group at 48 h (150.8 [115.2–151.6], 304.2 [240.2–331.6], vs. 87.6 [71.0–141.3], p < 0.05). Similarly, no significant difference was observed in AMPK, but an increase was observed in phosphorylation with the addition of Actinonin (431.2 ± 141.9 vs. 100.0 ± 69.7 or 110.3 ± 49.4, p = 0.013 or p = 0.015, respectively). A 50 µM dose of Actinonin increases PINK1 expression at 36 h, and at 48 h, the combination of si-GSK-3ß (15 nM) and Actinonin results in the highest PINK1 expression compared to si-control plus Actinonin or DMSO (232.9 ± 42.7 vs. 89.2 ± 4.2 or 100.0 ± 10.9, p = 0.001 or p = 0.002). PINK1 activates Parkin, which ubiquitinates mitochondrial outer membrane proteins, facilitating autophagy. However, Actinonin in both si-GSK-3ß and si-control leads to decreased Parkin expression at 36 and 48 h (69.3 ± 9.7 or 74.4 ± 5.4 vs. 100.0 ± 10.1, p = 0.009 or p = 0.034; 69.4 ± 9.6 or 77.4 ± 3.9 vs. 100.0 ± 9.5, p = 0.012 or p = 0.026). GSK-3ß interference reduces the phospho-Parkin ratio at 36 hours compared to Actinonin alone or DMSO (14.3 ± 3.3 vs. 76.0 ± 2.8 or 100.0 ± 17.3, p = 0.001 or p < 0.001). TOM20, important for mitochondrial protein transport, increases at 24 h, then decreases at 36 h, with si-GSK-3ß or si-control plus Actinonin significantly higher than the control at 24 h (250.5 ± 19.3 or 199.2 ± 24.7 vs. 100.0 ± 53.5, p = 0.005 or p = 0.034). At 36 h, significant decreases are observed (36.6 ± 2.6 or 56.1 ± 2.8 vs. 100.0 ± 13.2, p < 0.001 or p = 0.001). TFAM, a transcription factor involved in mtDNA transcription and replication, is upregulated by Actinonin at 36 h (149.0 ± 19.3 and 148.7 ± 21.9 vs. 100.0 ± 7.7, p = 0.032 and p = 0.033). Additionally, at 48 h, si-GSK-3ß plus Actinonin significantly elevates TFAM levels (218.4 ± 22.9 vs. 105.2 ± 13.5 or 100.0 ± 25.6, p = 0.001 or p = 0.002). Protein dynamics of PINK1, Parkin, TOM20, and other proteins localized to the mitochondrial membrane were evaluated after normalization based on mitochondrial quantity. Except for an increase in PINK1 at 48 h Actinonin addition, all parameters decreased compared to the control (Fig. S2A). Parkin and TOM20 showed behavior similar to that observed in conventional Western blots (Fig. S2A and Fig. 4B). These results indicate that Actinonin triggers intracellular energy depletion, resulting in heightened ER stress and DNA damage. The combination of si-GSK-3ß and Actinonin leads to significant mitochondrial damage but does not activate Parkin in PINK1/Parkin-dependent mitophagy.

### The combination of GSK-3ß interference and Actinonin affects aerobic respiration and glycolysis, leading to reduced tumor growth in the xenograft mouse model

**Fig. 5:**
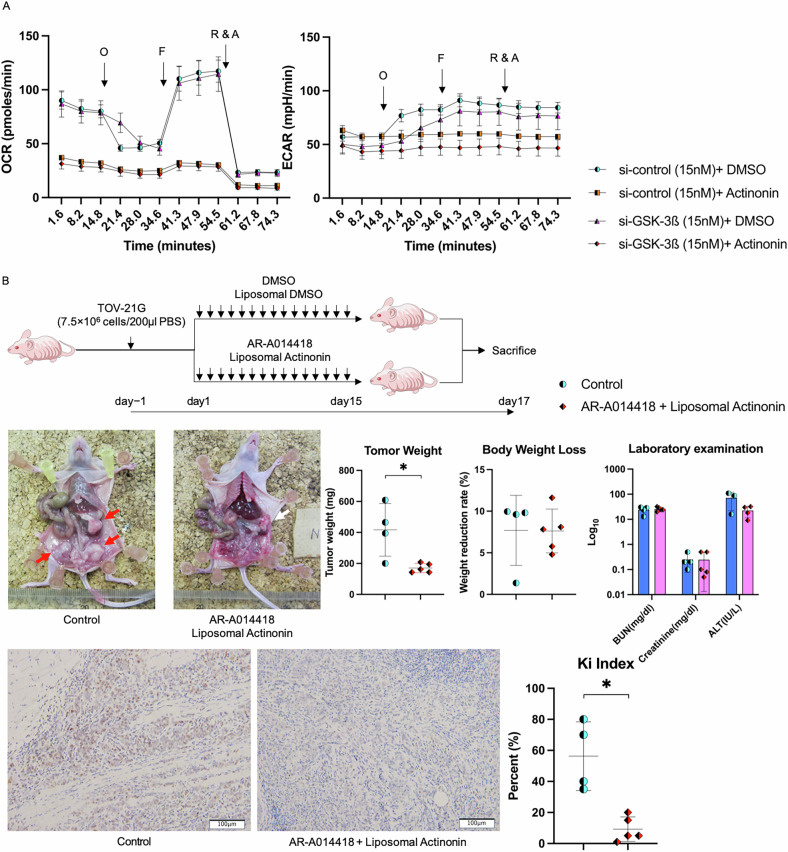
The combination of GSK-3ß interference and Actinonin affects aerobic respiration and glycolysis, leading to reduced tumor growth in the xenograft mouse model. **A** The experiment aimed to evaluate the aerobic respiration of mitochondria and glycolysis in four different combinations. The assessment was done by comparing the results with and without the interference of GSK-3ß (15 nM) and Actinonin (50 µM). After the rapid transfection of si-GSK-3ß or control (15 nM), Actinonin or DMSO was added at 24 h. The cells were then incubated for 24 h. (N = 3) **B** We conducted an in vivo assay using peritoneal dissemination in an athymic nude mice model. We combined DMSO in corn oil with liposomal DMSO as control (n = 4) or AR-A014418 with liposomal Actinonin as target (n = 5). We evaluated the tumor weight and body weight loss, and laboratory tests included BUN, creatinine, and ALT. The reagents were applied 15 times using a transperitoneal approach two days after abdominal tumor cell administration, after which the mice were sacrificed. And Ki-67 immunostaining was performed on tumor tissue samples collected to evaluate tumor proliferation capacity, and the Ki index was calculated. The error bars represent ±SD, and those displaying significant differences in normal distribution are marked with square brackets. Those displaying significant differences in non-normal distribution are marked with double arrows. GSK-3ß glycogen synthase kinase 3 beta, DMSO dimethyl sulfoxide, OCR oxygen consumption rate, ECAR extracellular acidification rate, O Oligomycin A, F carbonyl cyanide 4-(trifluoromethoxy) phenylhydrazone (FCCP), R & A, Rotenone/antimycin A. BUN, blood urea nitrogen, ALT alanine aminotransferase, Log logarithm; *, p < 0.05.

Actinonin (50 µM) significantly reduced basal respiration compared to the DMSO group (si-control [15 nM] and si-GSK-3ß [15 nM]) (90.0 ± 8.0 and 88.9 ± 4.2 vs. 36.9 ± 0.9 and 34.3 ± 1.7, p < 0.001)(Fig. 5A). It also decreased OCR and ECAR changes. After applying Rotenone/antimycin A, the ECAR levels for si-control and si-GSK-3ß under Actinonin differed significantly (57.3 ± 1.5 vs. 50.8 ± 1.6, p = 0.008)(Fig. 5A). Thus, Actinonin inhibits both mitochondrial respiration and glycolysis, though the baseline glycolysis in Actinonin alone is higher than with GSK-3ß interference. In vivo assay, the treatment was administered 15 times using DMSO in corn oil and liposomal formulations. The GSK-3ß inhibitor in combination with Actinonin significantly reduced total tumor weight in mice with disseminated tumors in the peritoneum, compared to controls (170.0 ± 29.7 vs. 417.0 ± 170.0, p = 0.014). Body weight reduction was similar between groups (7.8 [4.7–11.6] vs. 9.7 [1.3–9.9], p = 0.624), with no significant differences in liver or kidney function. Ki-67 expression was evaluated to assess cell proliferation. The GSK-3ß inhibitor in combination with Actinonin significantly reduced Ki-67 index compared to controls (9.2 ± 7.9 vs. 56.2 ± 22.1, p = 0.019)(Fig. 5B).

## Discussion

We have identified a novel antitumor agent that could positively impact HNF-1ß positive CCC. While Actinonin alone does not have an enough antitumor effect, its combination with GSK-3ß inhibitors significantly inhibits cell proliferation. Actinonin increases ROS stress in mitochondria, inducing mitophagy and potentially leading to immature mitochondria production, which disrupts aerobic respiration. The combination treatment fails to upregulate sufficient ROS scavengers like glutathione, causing ROS accumulation and mitochondrial damage. This is supported by increased transcription factors related to apoptosis, such as CHOP and GADD45 alpha. Overall, the combined treatment accelerates ROS accumulation and reduces glycolysis, leading to tumor cell apoptosis.

The N-terminal methionine excision (NME) pathway is essential for proper protein functioning in prokaryotic organisms. The formyl group is removed from the initial methionine in nascent peptides by PDF, and methionine aminopeptidase (MAP) subsequently removes the initial methionine [14]. It was previously believed that PDF existed only in prokaryotic organisms. However, recent research has discovered the presence of PDF and a MAP isoform in the mitochondria of eukaryotes, which raises questions about their role in human cells. Actinonin is a substance that inhibits PDF and was isolated from actinomycetes in 1962. It has been reported to have antimicrobial activity against both Gram-positive and biophilic Gram-negative microorganisms [15], then it has been clinically tested as a new anti-bacterial drug [16]. Moreover, a recent report suggests that Actinonin, a PDF inhibitor, may reduce aerobic respiratory capacity by influencing the expression of proteins derived from the mitochondrial DNA. This finding is significant as it sheds light on the potential therapeutic applications of Actinonin in treating mitochondrial diseases. Actinonin has potential benefits in treating obesity and Alzheimer’s disease by acting on mitochondria. It disrupts mitochondrial protein balance by inhibiting PDF, leading to increased fission, stress responses, and mitochondrial apoptosis [17]. Actinonin has been studied in various cancers, including colon, lung, leukemia, glioblastoma, and prostate cancer, in vitro and in vivo, and has been shown to be an effective chemotherapeutic compound [13, 16, 18–22]. Actinonin’s remarkable appearance belies its challenging usability due to low bioavailability, especially when administered in free form [23]. Then, some strategies have been devised, such as human serum albumin nanoparticles [16] and small gold nanoparticles (AuNP) [24]. We firstly administrated Actinonin in free form in vivo, but there was no significant tumor weight decrease, so we changed the application method. Improvement of the Actinonin delivery system should be considered for effective clinical trials in the future.

GSK-3 is a serine-threonine protein kinase that adds phosphate molecules to serine and threonine amino acid residues. It plays a key role in various cellular signaling pathways, including cell proliferation, cell migration, glucose regulation, and apoptosis. It has been identified as an important target in cancer treatment [25–31] as well as several neurodegenerative diseases, including Parkinson’s disease [32–34] and Alzheimer’s disease [35–39]. Some evidence has emerged that GSK-3ß may be involved in the glycolysis process, and inhibition of GSK-3ß showed decreased glycolysis and cell proliferation [26]. This may be from the inhibition of AMP-activated protein kinase (AMPK) and subsequent upregulation of the mammalian target of rapamycin (mTOR) [30, 40, 41]. Affecting PI3K/Akt/mTOR signaling pathway has been elucidated to be a molecular target to modulate glycolysis, as silencing of Glut1 on Akt/GSK-3ß/ß-catenin/survivin axis [42], an SGLT-2 inhibitor on signal transduction between PI3K/AKT/GSK-3ß/mTOR and Wnt/ß-catenin axis [43], and apatinib on VEGFR2/AKT1/SOX5/GLUT4 axis [44]. These glycolysis-modulating agents could be a reliable alternative method for GSK-3ß inhibitors.

This study has some limitations. The protein PINK1 accumulates at the outer membrane of mitochondria in response to damage or dysfunction. Then, the protein parkin is recruited from the cytosol to the outer mitochondrial membrane and phosphorylated by PINK1. Parkin’s E3 activity promotes mitophagy by tagging mitochondrial proteins with ubiquitin, which leads to their degradation. The use of Actinonin alone or in combination with GSK-3ß interference did not result in an upregulation of the parkin protein. This finding could provide valuable insight into the molecular mechanisms by which these agents function. It is still unclear how cells determine which energy production source to use, whether it be mainly glycolysis or oxidative phosphorylation. Identifying the key signal that governs this decision could lead to more effective energy usage. In conclusion, combining with GSK-3ß interference boosts ROS metabolism beyond tolerance limits, causing ROS to accumulate in lipid bilayers. This accumulation disrupts mitochondrial regeneration, forcing tumor cells to stop mitochondrial renewal and cell proliferation, ultimately leading them toward apoptosis.

## Supplementary information

Supplementary Figure 1

Supplementary Figure 2

Supplementary Figure Legends

Western blot images

Inhibitor Screening Result

## Data Availability

The data produced in this study is available for download at 10.5281/zenodo.13932329.
