# Supplementary figures and images for: Inhibition of glycolysis and stimulation of mitochondrial biogenesis lead to increased ROS levels and cell death in HNF-1ß positive clear cell carcinoma
Source: Cell Death Dis. 2025 Dec 1;16(1):879. doi: 10.1038/s41419-025-08243-2 (PMC12669797; doi:10.1038/s41419-025-08243-2)

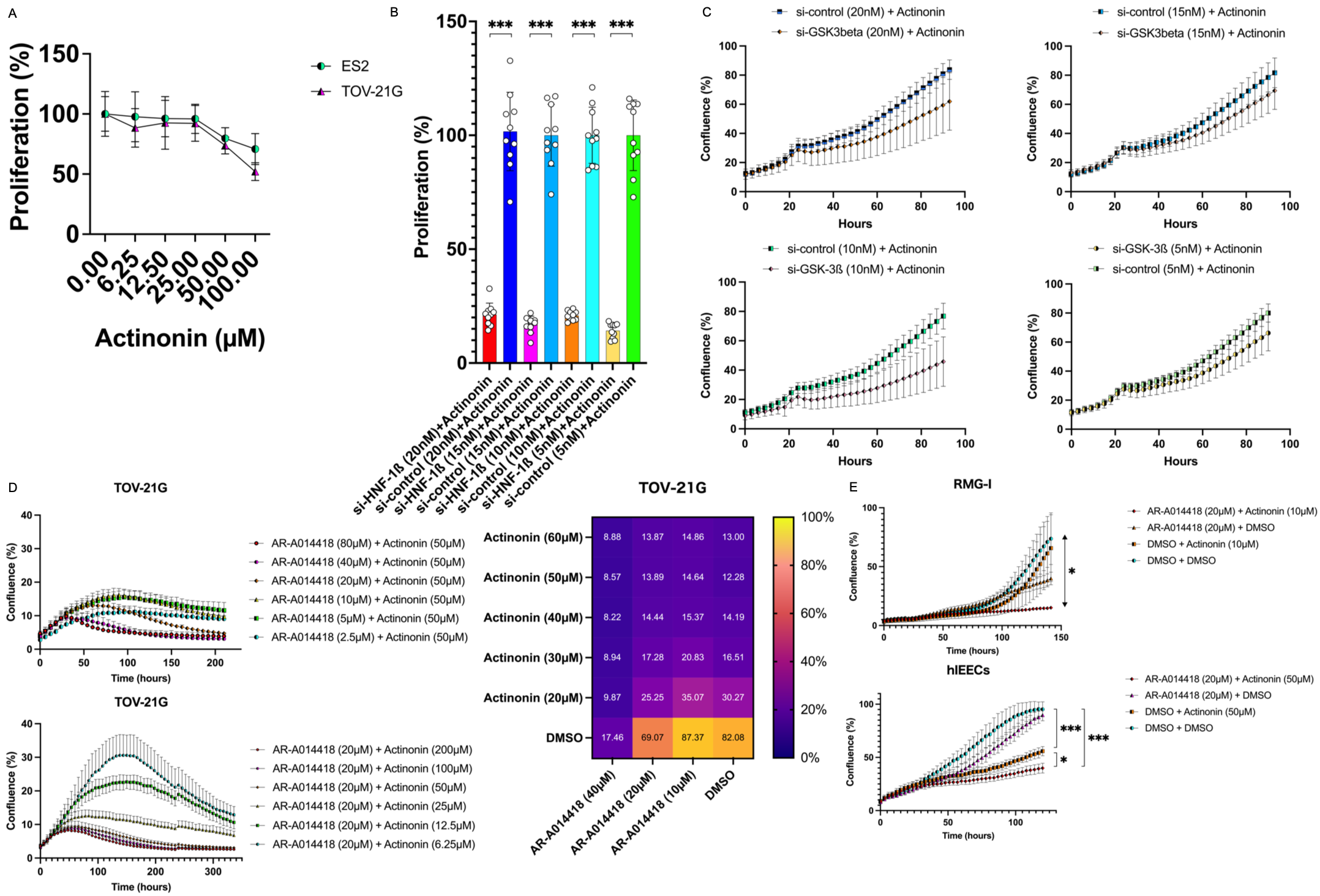

Supplement: Supplementary file 1 — Supplementary Figure 1 [file 41419_2025_8243_MOESM1_ESM.tif]

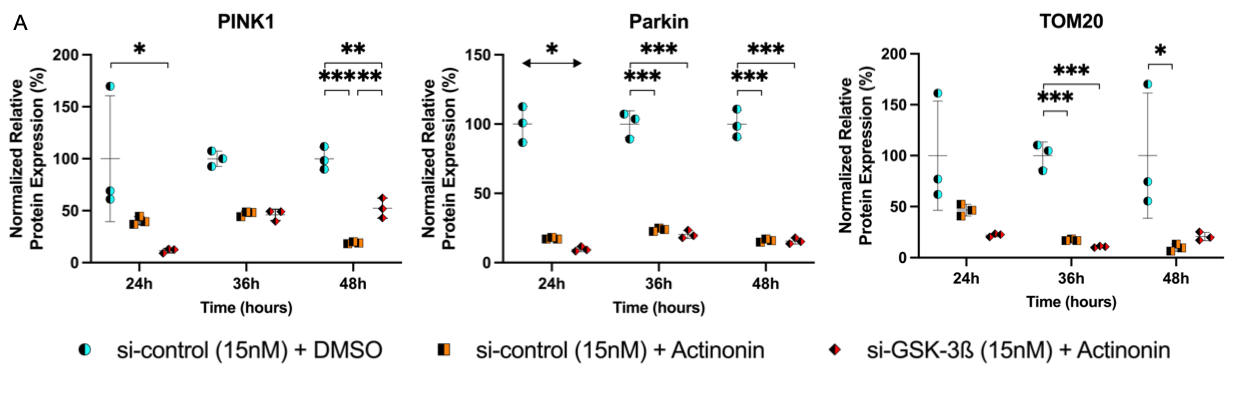

Supplement: Supplementary file 2 — Supplementary Figure 2 [file 41419_2025_8243_MOESM2_ESM.tif]

m-TOR

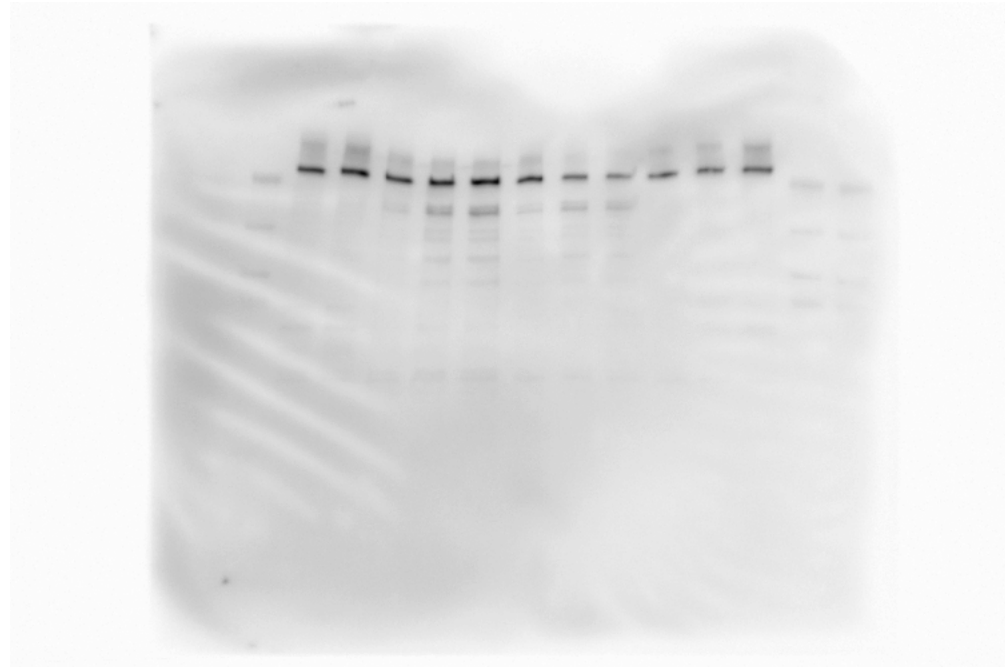

p-mTOR

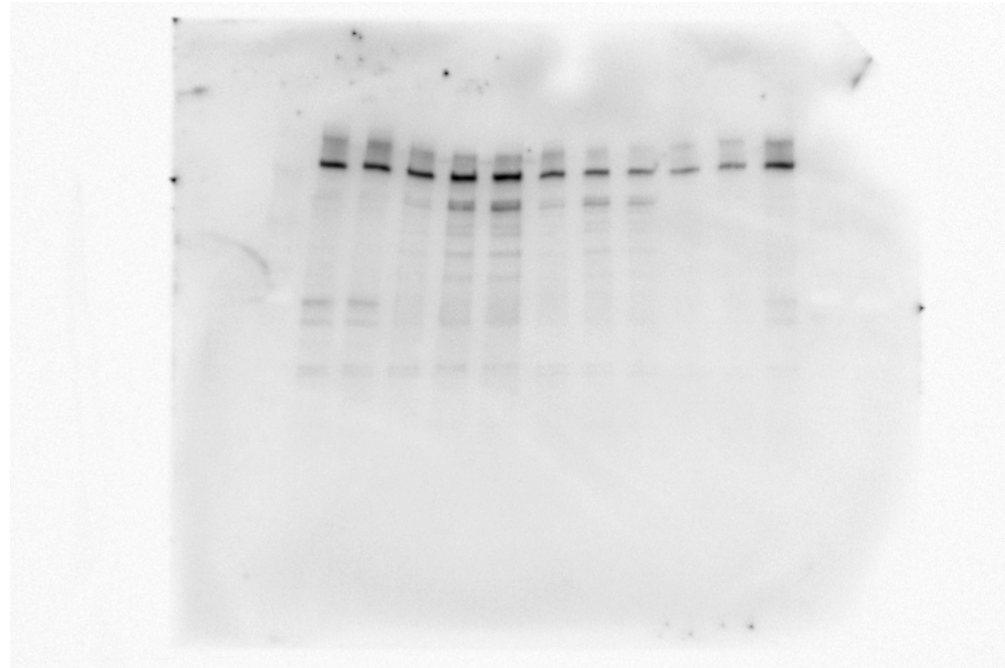

# AMPK

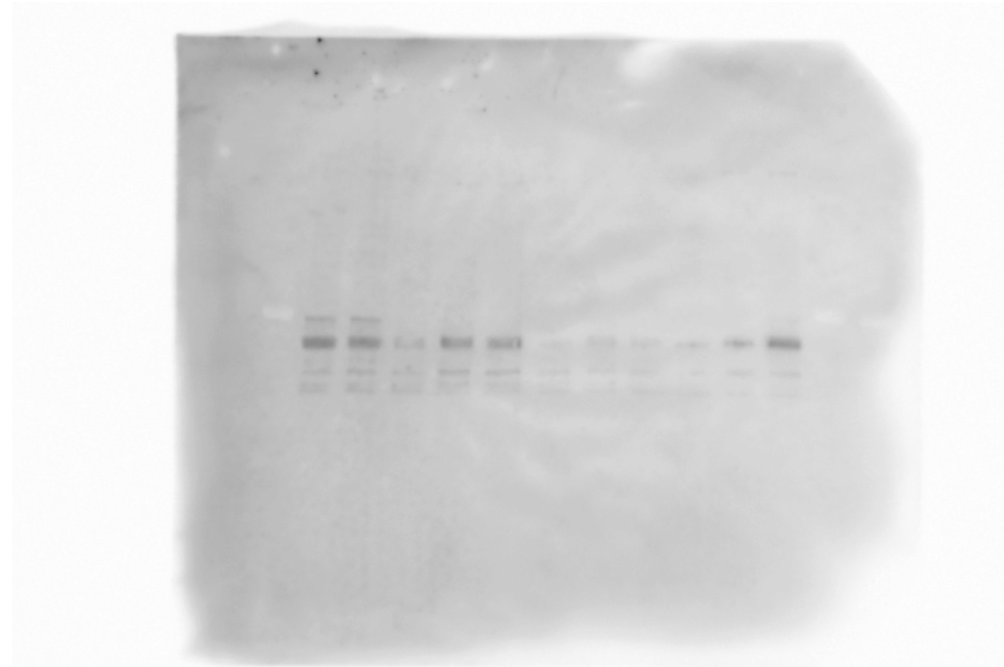

p-AMPK

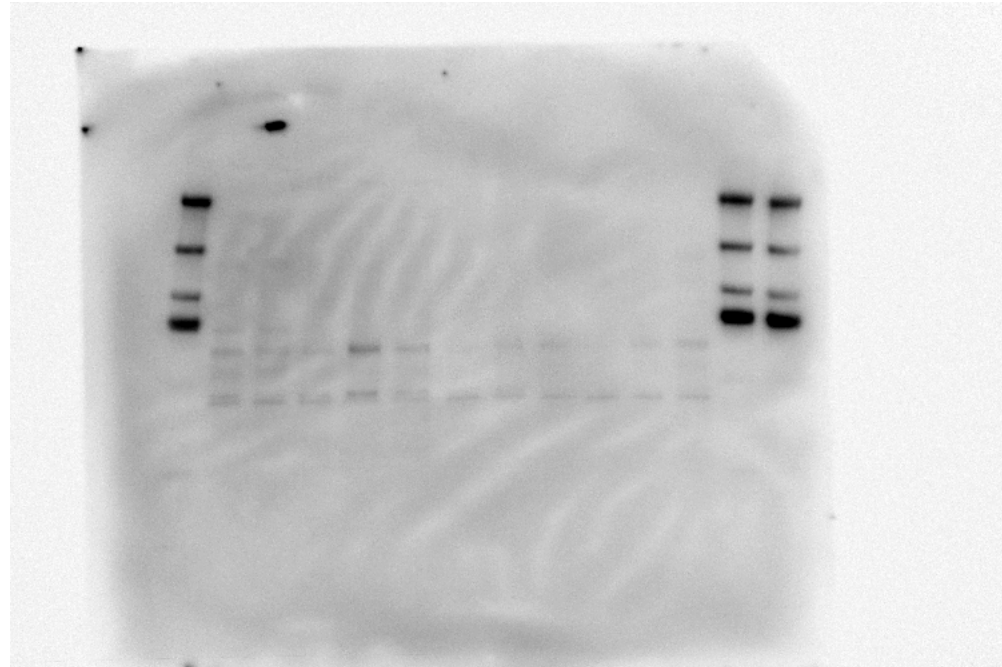

GSK-3 $\beta$

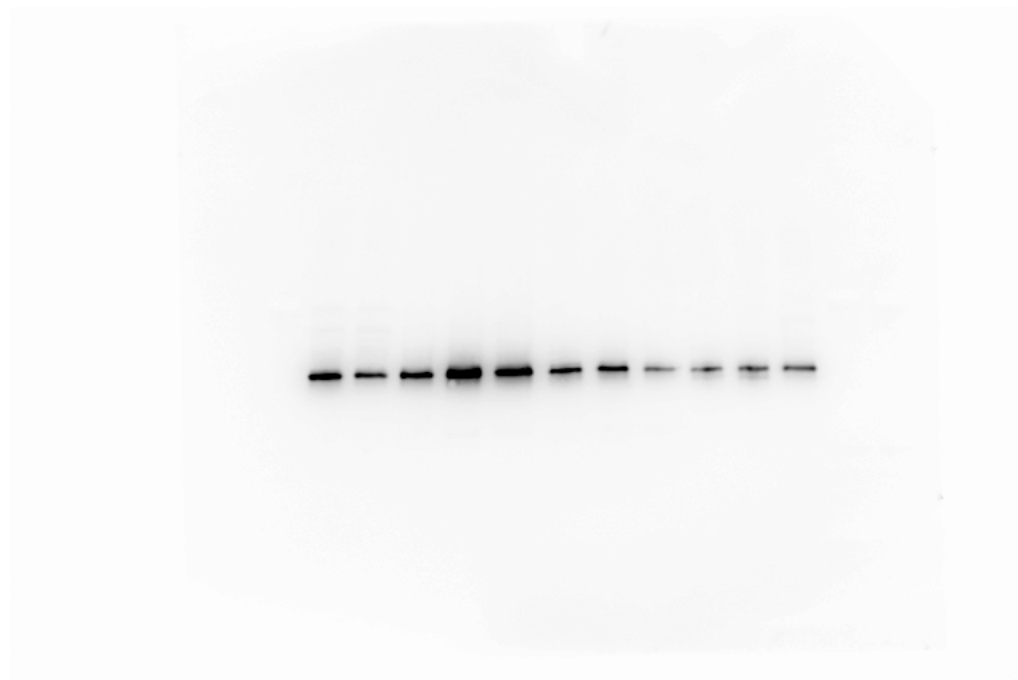

PINK1

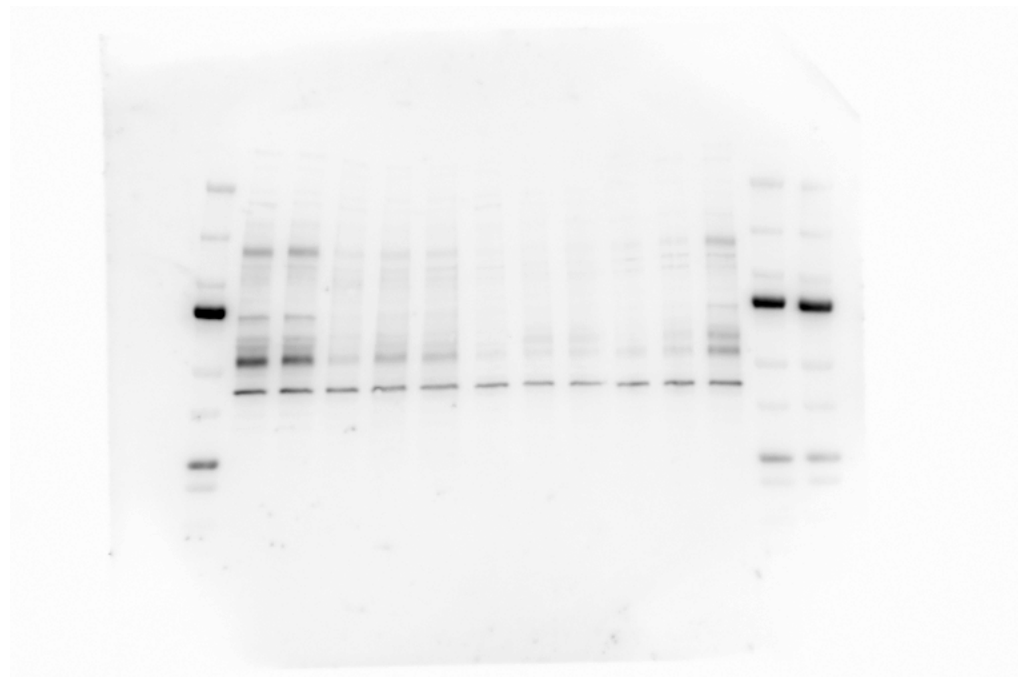

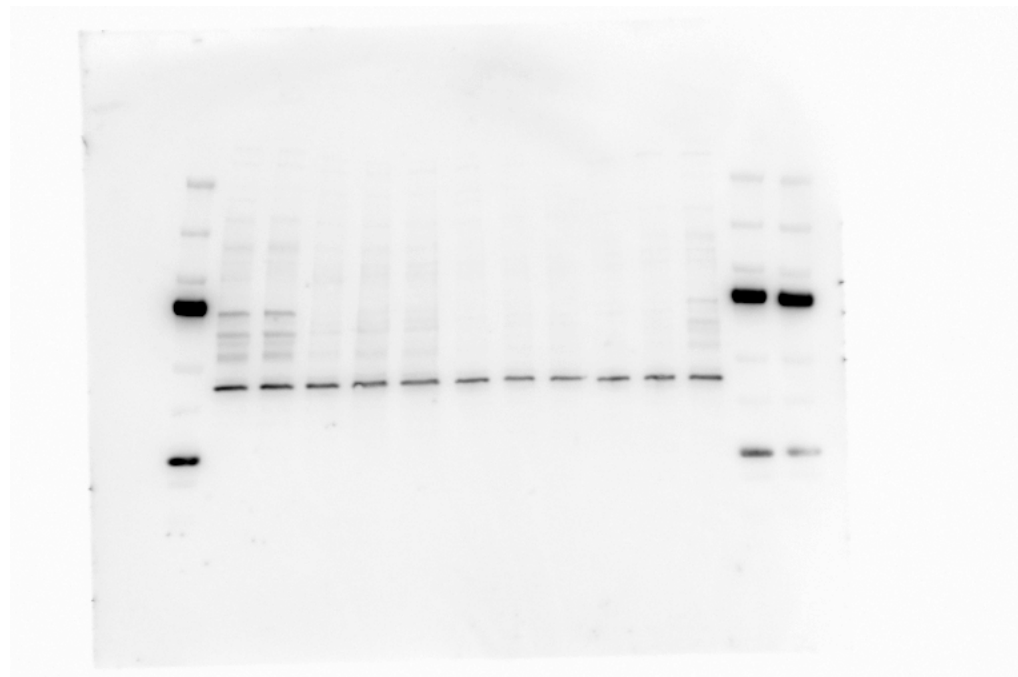

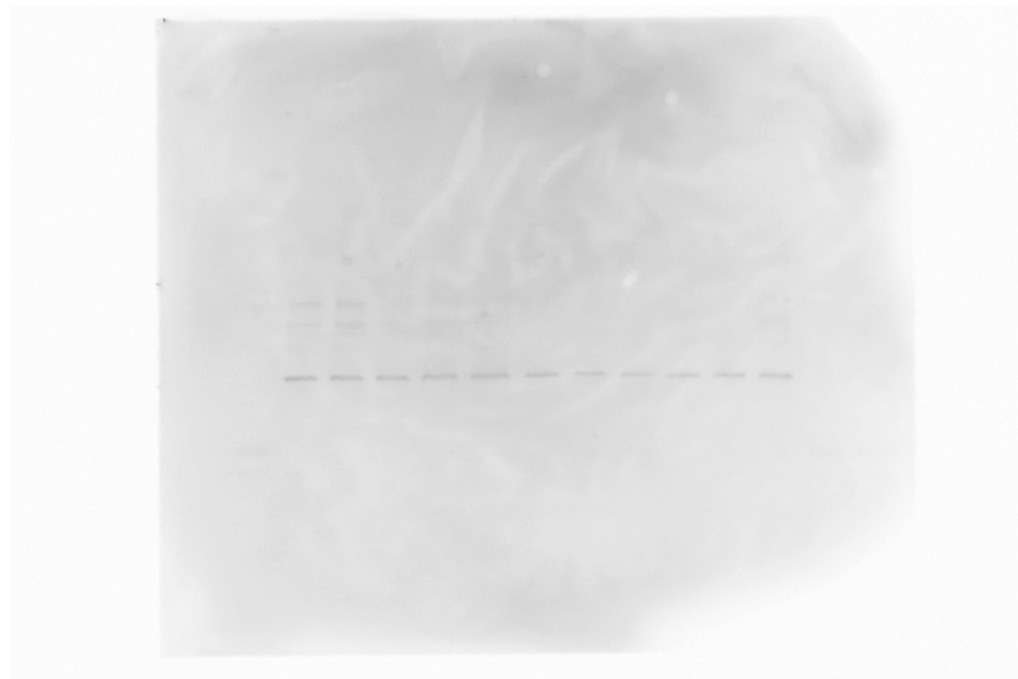

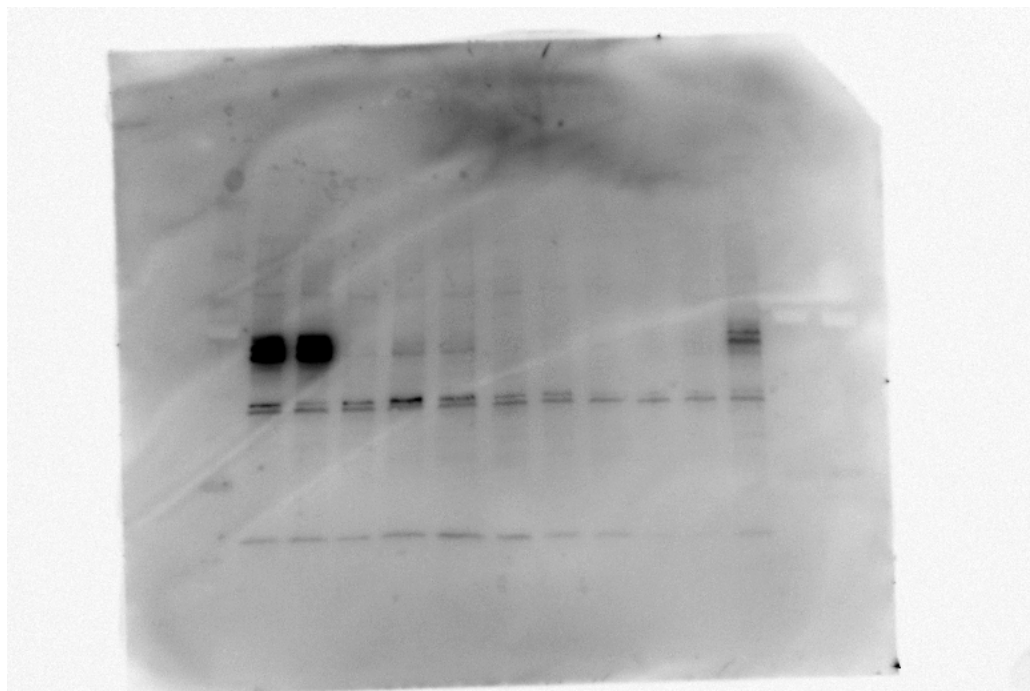

TFAM

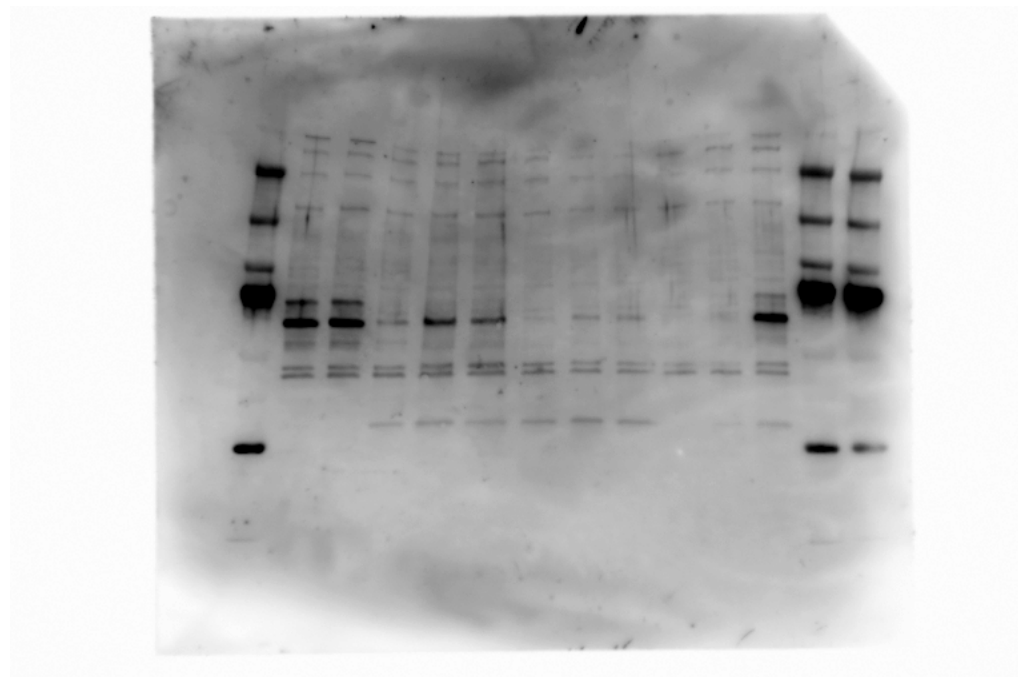

$\beta$ -actin

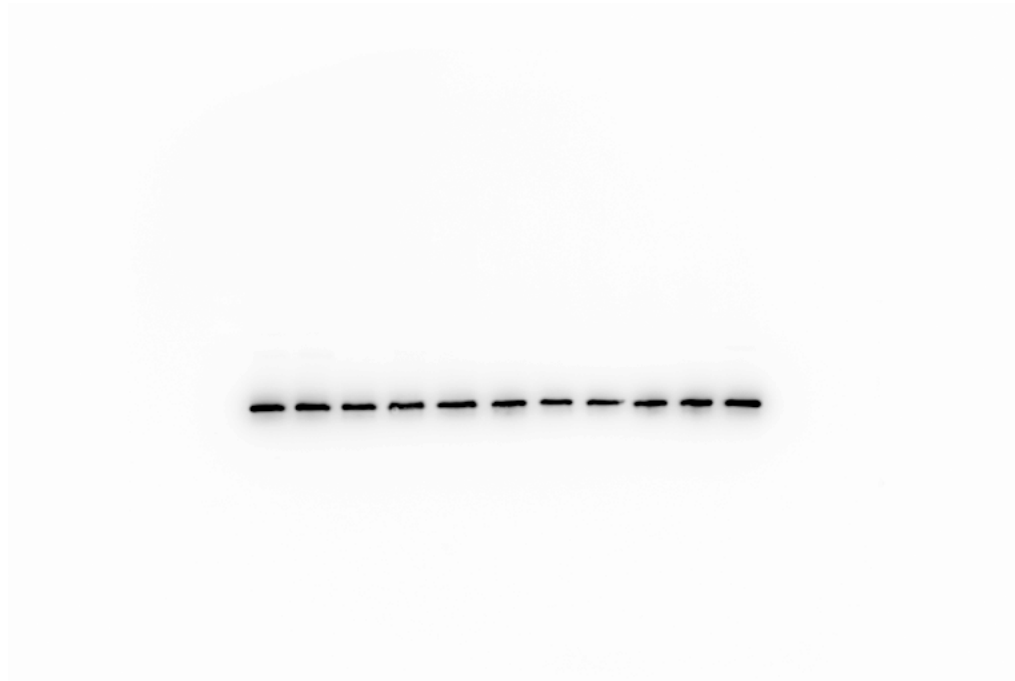

Supplement: Supplementary file 4 — Western blot images [file 41419_2025_8243_MOESM4_ESM.pdf]
