# Supplementary material for: Inhibition of glycolysis and stimulation of mitochondrial biogenesis lead to increased ROS levels and cell death in HNF-1ß positive clear cell carcinoma
Source: Cell Death Dis. 2025 Dec 1;16(1):879. doi: 10.1038/s41419-025-08243-2 (PMC12669797; doi:10.1038/s41419-025-08243-2)
Supplement: Supplementary file 3 — Supplementary Figure Legends [file 41419_2025_8243_MOESM3_ESM.docx]

**Supplementary Figure 1.** The mild effects on HNF-1ß negative cell line by GSK-3ß interference plus Actinonin.

(A) Dose-response curve for TOV-21G, which is HNF-1ß-positive, and ES2, which is HNF-1ß-negative. (N=10) (B) Even slight interference with HNF-1ß, which is upstream of GSK-3ß, strongly promotes the effect of Actinonin addition. (N=10) (C) ES2, a negative HNF-1ß cell line, initially inhibited proliferation, but this effect quickly diminished due to GSK-3ß interference with Actinonin at all concentrations. (N=3) (D) The antitumor effect of combining GSK-3ß inhibitor with Actinonin produced similar results at all concentrations (N=10), and the antitumor effect of combining GSK-3ß inhibitor with Actinonin produced similar results at all concentrations. (N=3) (E) We also verified that Actinonin replicates GSK-3ß inhibition in RMG-I, a clear cell carcinoma cell line expressing HNF-1ß. Conversely, there was no impact on cell proliferation in human immortalized epithelial cells (hIEECs). (N=3)

The error bars represent ±SD, and those displaying significant differences in normal distribution are marked with square brackets. Those displaying significant differences in non-normal distribution are marked with double arrows. HNF-1ß, hepatocyte nuclear factor-1 beta; GSK-3ß, glycogen synthase kinase 3 beta; *, p < 0.05; ***, p < 0.001.

**Supplementary Figure 2.** Protein evaluation corrected for intracellular mitochondrial content.

(A) Normalization based on mitochondrial mass for PINK1, Parkin, and TOM20. The addition of Actinonin reduces PINK1 and Parkin, and TOM20 also decreases similarly. (N=3) The error bars represent ±SD, and those displaying significant differences in normal distribution are marked with square brackets. Those displaying significant differences in non-normal distribution are marked with double arrows. PINK1, PTEN-induced putative kinase 1; TOM20, Translocase of outer mitochondrial membrane 20; DMSO, dimethyl sulfoxide; *, p < 0.05; **, p < 0.01; ***, p < 0.001.
